# Loss of Fertility in the Absence of Progesterone Receptor Expression in Kisspeptin Neurons of Female Mice

**DOI:** 10.1371/journal.pone.0159534

**Published:** 2016-07-21

**Authors:** Arnon Gal, Po-Ching Lin, Joseph A. Cacioppo, Patrick R. Hannon, Megan M. Mahoney, Andrew Wolfe, Rodrigo Fernandez-Valdivia, John P. Lydon, Carol F. Elias, CheMyong Ko

**Affiliations:** 1 Institute of Veterinary, Animal and Biomedical Sciences, Massey University, Palmerston North, New Zealand; 2 Department of Comparative Biosciences, College of Veterinary Medicine, University of Illinois at Urbana-Champaign, Urbana, IL, United States of America; 3 Department of Pediatrics, Johns Hopkins University School of Medicine, Baltimore, MD, United States of America; 4 Department of Pathology and Department of Oncology, Wayne State University School of Medicine, Detroit, MI, United States of America; Tumor Biology Microenvironment Program, Karmanos Cancer Institute, Detroit, MI, United States of America; 5 Center of Reproductive Medicine and Department of Molecular and Cellular Biology, Baylor College of Medicine, Houston, TX, United States of America; 6 Department of Molecular and Integrative Physiology, University of Michigan, Ann Arbor, MI, United States of America; The University of Georgia, UNITED STATES

## Abstract

Ovarian steroids, estradiol and progesterone, play central roles in regulating female reproduction by acting as both positive and negative regulators of gonadotropin-releasing hormone (GnRH) secretion in the hypothalamus. Recent studies have identified kisspeptin neurons of the hypothalamus as the target of estrogenic regulation of GnRH secretion. In this study, we aimed to determine the significance of progesterone receptor (PGR) expression in the kisspeptin neurons. To this end, the *Pgr* gene was selectively ablated in mouse kisspeptin neurons and the reproductive consequence assessed. The hypothalamus of the *Pgr* deficient female mouse expressed kisspeptin, the pituitary released LH in response to GnRH stimulation, and the ovary ovulated when stimulated with gonadotropins. However, the mutant mouse gradually lost cyclicity, was unable to generate a LH surge in response to rising estradiol, and eventually became infertile. Taken together, these results indicate that the loss of PGR impairs kisspeptin secretory machinery and therefore that PGR plays a critical role in regulating kisspeptin secretion.

## Introduction

The ovarian steroids estrogen and progesterone regulate the central female reproductive axis [1, 2]. Estrogen provides positive feedback to the hypothalamus in the late follicular phase that is necessary for the induction of the GnRH-mediated preovulatory luteinizing hormone (LH) surge, whereas during the rest of reproductive cycle it exerts negative feedback on the release of GnRH [2, 3]. However, the molecular mechanism for this hormonal switch remains to be fully determined [3]. Similar to estrogen, progesterone is necessary for the induction of the preovulatory LH surge [1], but otherwise exerts negative feedback on GnRH release during the rest of the estrous cycle [4–6]. Estradiol and progesterone feedback regulation of the hypothalamus is, in part, mediated through kisspeptin neurons [7]. Once released from kisspeptin neurons, this neuropeptide stimulates GnRH secretion via its receptor, *Gpr54 (Kiss1r)*, that is present on GnRH neurons [8]. Recent studies showed that the ablation of either the *Kiss1* or *Gpr54* gene in mice results in severe hypogonadism and subsequently infertility [9]. Similarly, spontaneous mutation in the human kisspeptin receptor gene, *GPR54*, results in severe infertility (hypogonadotropic hypogonadism) [10].

In rodents, two subpopulations of kisspeptin neurons are present at distinct anatomic locations: the anteroventral periventricular nucleus (AVPV) and the arcuate nucleus (ARC) [11], where estrogen regulates *Kiss1* expression via its receptor ERα (ESR1) [12]. *Kiss1* expression in these neuronal subpopulations is regulated differently: estrogen stimulates *Kiss1* expression in the AVPV and down regulates it in the ARC [11, 12]. Exposure to estrogen is required for the expression of the *Kiss1* gene in AVPV kisspeptin neurons [13], and kisspeptin is crucial for GnRH release [9]. Kisspeptin neurons express PGR [9] as well as ERα (*Esr1*). Because estrogen and progesterone exert both positive and negative feedback regulation on the hypothalamus [1–6] and PGR expression is regulated by estrogen in a number of estrogen responsive tissues [14–16], PGR expression is likely under ERα regulation and is required for estrogen action in the kisspeptin neurons. In this study, we tested the hypothesis that PGR expression in kisspeptin neuron is required for fertility.

## Materials and Methods

### Generation of *Kiss1-PgrKO*, global *Pgr*, and global *ER*α knockout mice

All mice used were of the C57BL/6 genetic background, bred by the University of Illinois Division of Animal Resources and were maintained under controlled lighting (14 h light/10 h dark) with continuous access to food and water. The *Pgr* gene was either selectively ablated in kisspeptin neurons or globally, by crossbreeding floxed *Pgr* mice (*Pgr*^*flox/flox*^) [17] with transgenic mice that express Cre recombinase under the *Kiss1* promoter (Kiss1Cre) [18] or under the *Zp3* promotor (Zp3Cre) [19], respectively. Mice with the genotype *Pgr*^*flox/flox*^*Kiss1*-Cre were generated by successive back crossing of F1 generation mice with *Pgr*^*flox/flox*^ mice, and are named herein as Kiss1-PgrKO (kisspeptin neuron specific *Pgr* knockout). The kisspeptin neuron-specific *Pgr* gene deletion was confirmed by double immunohistochemistry with anti-Pgr antibody (A0098, Dako, Carpinteria, CA, USA) and anti-kisspeptin antibody (AB9754, Millipore, Billerica, MA, USA). Mice with the genotype *Pgr*^-/-^ (global Pgr knockout; G-PgrKO) were generated by successive back crossing of F1 generation mice from *Pgr*^*flox/flox*^ and *ZP3Cre* litters. *Pgr* gene deletion was similarly confirmed by immunohistochemistry with an anti-Pgr antibody. Mice with the genotype *Esr1*^-/-^ (global ERαknockout; ERαKO) were generated by successive back crossing of F1 generation mice from *Esr1*^*flox/flox*^ and *ZP3Cre* litters. *Esr1* gene deletion was confirmed by immunohistochemistry with an anti-ERα antibody (sc-542, Santa Cruz, Santa Cruz, CA, USA). Mice were housed at the University of Illinois at Urbana-Champaign (UIUC) animal-care facility under 12 hour light/dark cycles. Animals were fed commercial rodent diet and had free access to water. This study was approved by the University of Illinois at Urbana-Champaign Institutional Animal Care and Use Committee.

Mouse genotypes were determined by PCR. Tissue from ear pinna was obtained by an ear punch and DNA was extracted with an Easy DNA kit (Invitrogen, Carlsbad, CA, USA) according to the manufacturer’s instructions. All of the following PCR reactions were done with a total reaction volume of 10 μl with 5 μl of Bioline Mango mix (Bioline, Taunton, MA, USA), 1 μg of genomic DNA, 0.2 μM of each primer, and double distilled water to bring the reaction volume to 10 μl. Three primers were used to amplify the floxed *Pgr* gene: fPR-F (5’-GTATGTTTATGGTCCTAGAGCTGGG-3’), fPR-R (5’-TGCTAAAGGTCTCCTCATGTATTGGG-3’), and fPR-DR (5’-ATATTTATGACTTTGAGACTTG-3’). The expected band sizes for WT, floxed *Pgr*, and *Kiss1-PgrKO* genotypes were 226 bp, 276 bp and 481 bp, respectively. Two primers were used for amplifying the Cre sequence [18]: M247 (5’-TGCGAACCTCATCACTCGTTGCAT-3’) and M358 (5’-GCTCTGGTGAAGTACGAACTCTGA-3’). Expected band sizes for mice carrying the Cre gene and WT mice were 189 bp and 330 bp, respectively. PCR conditions for amplifying the flox sequence in the *Pgr* gene were an initial denaturation at 94°C for 5 minutes (min) followed by 30 cycles of: denaturation at 94°c for 1 min, annealing at 58°C for 1 min, and extension at 72°C for 2 min. The PCR terminated with a final extension step at 72°C for 10 min. PCR conditions for amplifying the Cre sequence in the *Kiss1* gene were an initial denaturation at 94°C for 4 min followed by 35 cycles of: denaturation at 94°C for 30 s, annealing at 58°C for 30 s, and extension at 72°C for 45 s. The PCR reaction was terminated with a final extension step at 72°C for 7 min. PCR amplicons were run on a 2% agarose gel with GelRed (Biotium, Hayward, CA, USA) at 100 mV for 25 min and visualized under UV light.

### RNA extraction, cDNA synthesis, and qPCR

Total RNA was extracted from AVPV (plate 30 [20]) tissue by a 0.5 mm Harris Uni-Core punch biopsy (Sigma-Aldrich, St. Louis, MO, USA) and an RNAqueous^®^-Micro Kit (Ambion^®^ by Life Technologies^™^, Carlsbad, CA) according to the manufacturer’s protocol; concentrations of RNA were measured with a NanoDrop 1000 spectrophotometer (Thermo Scientific, Asheville, NC) and diluted to equal concentrations. RNA was reverse transcribed using a high capacity cDNA reverse transcription kit (Applied Biosynthesis, Foster city, CA). PCR reactions were performed with TaqMan^®^ universal PCR master mix (Applied Biosynthesis, Foster city, CA), with the *Kiss1* primer/probe set (Mm03058560_m1) designed to span the intron between the 2 coding exons of the *Kiss1* gene and L19 primer/probe set (Mm02601633_g1). Fluorescence was measured using the ABI prism 7500 quantitative real-time thermocycler (Applied Biosystems). Each reaction was run in triplicate. Fold changes in relative gene expression were calculated by 2^Δ(ΔCt), where ΔCt = Ct (Kiss1) − Ct(L19) and Δ(ΔCt) = ΔCt (Kiss1-PgrKO) − mean ΔCt (WT) [21]. Results are expressed as fold differences in relative gene expression with respect to WT.

### Tissue handling & processing, hematoxylin & eosin staining, immunohistochemistry & immunofluorescence

At any endpoint in the study, mice were humanely euthanized by CO_2_ asphyxia followed by cervical dislocation. Collected tissue was immediately fixed in 4% paraformaldehyde for 24 hours. Tissues were washed with ethanol, processed, and embedded in paraffin wax. Paraformaldehyde-fixed paraffin-embedded tissue blocks were sectioned 5 μm thick in a series so that consecutive 10^th^ sections were mounted on the same glass slide.

Immunolabeling of PGR, ERα and kisspeptin was done in the following manner: deparaffinization was followed by heat-induced antigen retrieval in 10 mM sodium citrate buffer (pH 6.0), endogenous peroxide quenching in 3% H_2_O_2,_ and antibody and endogenous biotin blockage by 5% goat serum with avidin (200 μl/1ml; Avidin/biotin blocking kit SP-2001, Vector labs, Burlingame, CA, USA). Incubation of the primary rabbit anti-mouse PGR antibody (A0098, Dako, Carpinteria, CA, USA) and biotin (200 μl/1ml; Avidin/biotin blocking kit SP-2001, Vector labs, Burlingame, CA, USA) was followed by incubation with a secondary biotinylated goat anti-rabbit antibody (Vectastain ABC kit, Vector labs, Burlingame, CA, USA) and avidin biotin complex solution (Vectastain Elite ABC kit, Vector labs, Burlingame, CA, USA) at room temperature. 3,3’-diaminobenzidine Nickel (DABN; SK-4100, Vector labs, Burlingame, CA, USA) was applied until color optimally developed. Tissues were re-blocked with goat serum followed by incubation with a rabbit polyclonal ERα antibody (MC-20, Santa Cruz, Dallas, Texas, USA) and biotin, and subsequent incubation with a secondary goat anti-rabbit antibody. Tissues were once again re-blocked with goat serum and avidin followed by incubation with a rabbit anti-mouse kisspeptin antibody (AB9754, Millipore, Billerica, MA, USA) and biotin, and subsequent incubation with a secondary biotinylated goat anti-rabbit antibody, and incubation with an avidin biotin complex solution. 3,3’-diaminobenzidine (DAB) was applied until optimal color development. Slides were then rinsed, counter-stained and cover slipped.

Immunofluorescent labeling was performed similarly to immunohistochemistry with the following modifications: 1) the endogenous peroxide blocking step was omitted; 2) the ABC complex step was omitted and instead the sections were incubated with one of the following fluorophores: Alexa Fluor^®^594-conjugated Streptavidin, Alexa Fuor^®^488-conjugated Streptavidin, or Alexa Fuor^®^647-conjugated Stretavidin (Jackson ImmunoResearch, West Grove, PA).

### Fertility assay and counting of follicles, corpora lutea, and neurons

Fertility of 3 and 5 month old wild-type (WT) and Kiss1-PgrKO mice was determined by housing together a proven breeder male with one Kiss1-PgrKO and one WT female for 10 days. Cages were inspected daily for presence and size of a litter. The final outcome of the fertility test was determined after 30 days. Percent fertility was calculated as the number of females that gave birth divided by the total number of females in the group. Follicle and CL counting were done as previously described [22–24], and performed by one of the authors (PRH) who was blinded to the source of the slides. PGR and/or kisspeptin neuron counting was done by a single author (AG) who was blinded to the source of the slides. Raw cell number was calculated from the sum of immunolabeled cells in 3 AVPV (plate 30 [20]) sections that were 50 μm apart.

### Serum gonadotropin measurement

Serum luteinizing hormone (LH) and follicle-stimulating hormone (FSH) levels were measured by Luminex assay [25]. The intra-assay coefficient of variations (CVs) for LH and FSH were between 0.7%-4.4% and 2%-9%, respectively. The inter-assay CVs for LH and FSH were 3.7%-7.8% and 4.3%-6.8%, respectively. The assay detection limit for LH was 12.2 pg/ml and for FSH 61 pg/ml. No samples were below the detection limit of the assay.

### Stimulation tests with estradiol, GnRH and gonadotropins

Five-month-old mice were superovulated by intraperitoneal injection of 5 IU pregnant mare's serum gonadotropin (PMSG) and, 48 h later, the mice were additionally injected with 5 IU human chorionic gonadotropin (hCG). The mice were euthanized 22 h after the injection of hCG for quantification of ovulated oocytes as previously described [26]. GnRH stimulation was performed in 5-month-old WT and Kiss1-PgrKO by intraperitoneal injection of 200 ng/kg GnRH peptide (Sigma–Aldrich) in 100 μl saline. Blood was collected 10 min later via cardiac puncture immediately following euthanasia [27]. Estrogen treatment was performed as previously described [28]. Briefly, mice were ovariectomized, and 1 cm SILASTIC (Dow Corning Corp., Midland, MI) brand capsules (0.04”ID, 0.085” OD; American Scientific Products, McGaw Park, IL) containing 2.5 μg of 17β-estradiol (17β-E2, Sigma Chemical Co., St. Louis, MO) mixed into Silicone Type A Medical Adhesive (Dow Corning Corp.) were placed subcutaneously under one flank. Controls were implanted with empty capsules. Following 6 days, mice were injected subcutaneously with estradiol benzoate (EB, 1 μg in 0.1 ml sesame oil) or vehicle at 0900 h. On the evening of the seventh day following ovariectomy at 1900 h, blood was collected via cardiac puncture immediately following euthanasia.

### Statistical data analyses

Data analyses were performed using statistical software (SPSS, New York, NY, USA). All normally distributed continuous data were analyzed with parametric tests (t-test or one-way ANOVA) and a post hoc test (Tukey). All non-normally distributed continuous data were analyzed with nonparametric tests (Mann-Whitney and Kruskal Wallis). Normally distributed data are presented as mean and standard deviation, whereas non-normally distributed data are presented as median and range. For all analyses the alpha value was set to 0.05.

## Results

### Loss of *Pgr* in kisspeptin neurons alters estrous cyclicity, fertility and serum LH concentration

**Fig 1:**
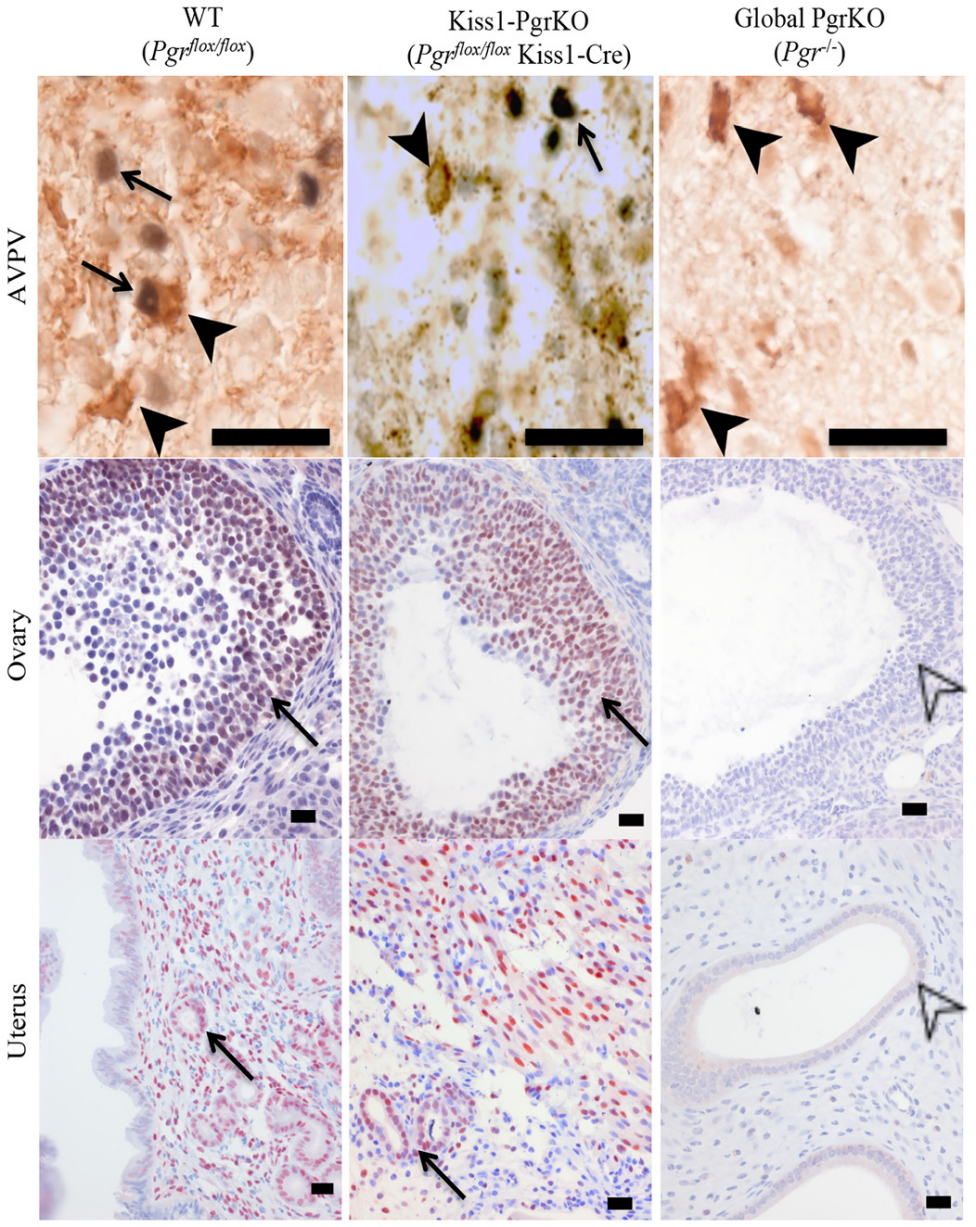
Immunohistochemical labeling of PGR and kisspeptin in neurons from the AVPV, and cells from the ovary and uterus of 2-3-month-old WT (left), Kiss1-PgrKO (middle), and global-PgrKO (right). Black arrows indicate PGR positive nuclei, black arrowheads indicate cytoplasmic kisspeptin, open arrowheads indicate nuclei that do not express PGR. Bar in AVPV = 20 μm; bar in uterus and ovary = 25 μm.

The *Pgr* gene was selectively ablated by crossing the floxed *Pgr* mouse strain with a transgenic mouse strain that expresses Cre recombinase under the *Kiss1* promoter [18, 19]. Immunohistochemistry was used to determine if *Pgr* expression was ablated in kisspeptin neurons of the resulting double transgenic strain of mice (*Pgr*^*flox/flox*^;*Kiss1Cre*). In the female Kiss1-PgrKO mouse, PGR expression was obvious in the uterus and ovaries, but not detectable in the kisspeptin neurons (Fig 1).

**Fig 2:**
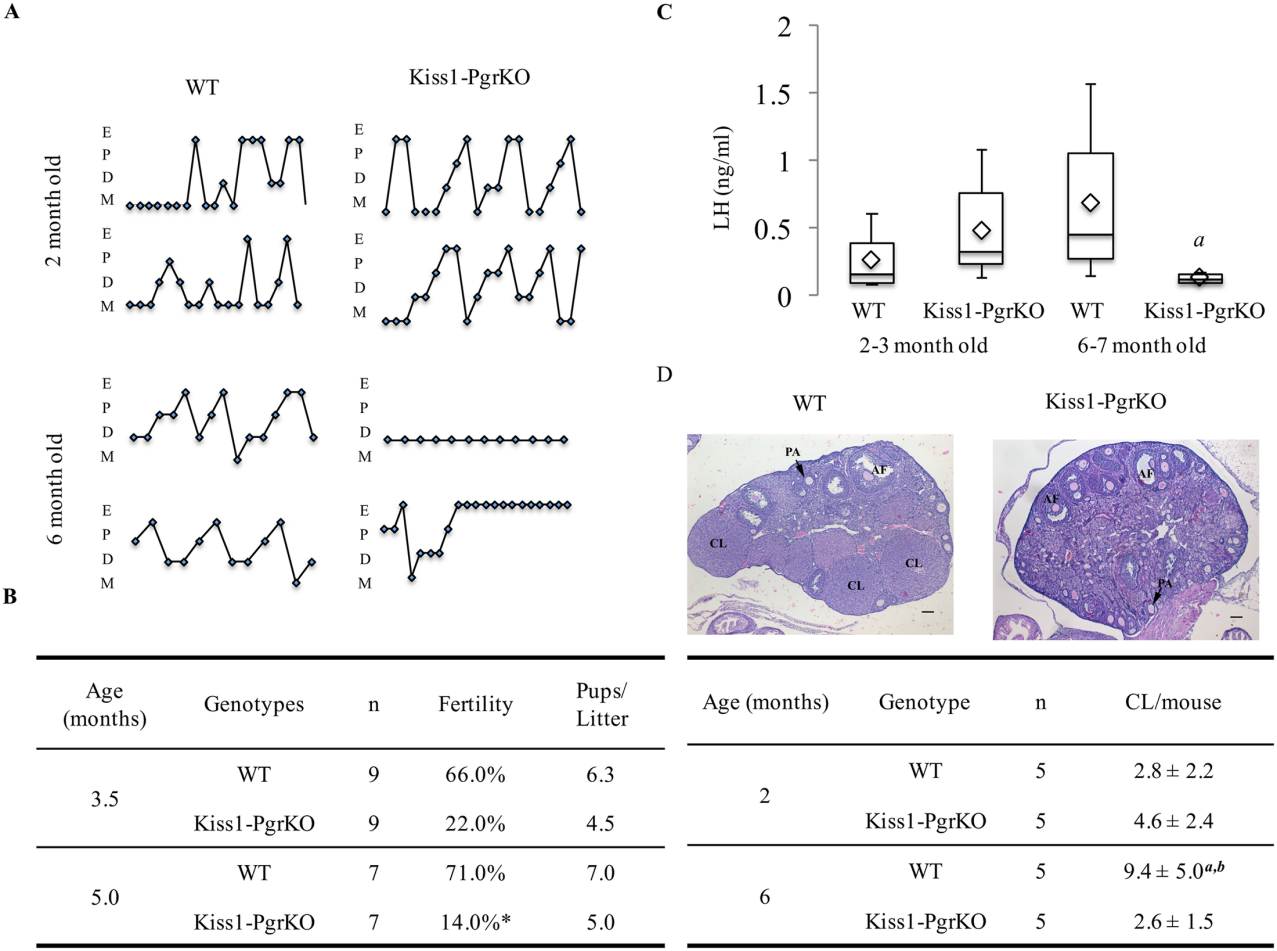
Loss of Pgr in kisspeptin neurons leads to altered cyclicity, progressively impaired fertility, decreased serum LH, and absence of corpora lutea formation. A. Two representative estrous-cycle cycling patterns from 1.5- and 6-month-old WT and Kiss1-PgrKO mice. B. Fertility assay results for 3.5- and 5-month-old WT and Kiss1-PgrKO mice (asterisk represents *p* = 0.03). C. Box plot data of diestrus serum LH concentration of 2-3-month-old WT (n = 5) and Kiss1-PgrKO (n = 10) mice and 6-7-month-old WT (n = 6) and Kiss1-PgrKO (n = 4) mice; diamonds represent the mean. *a* represents difference between 6–7 moth old WT and Kiss1-PgrKO mice (*p* = 0.024). D. Representative images of 6-month-old WT and Kiss1-PgrKO ovaries (n = 5); CL–corpus luteum, AF–antral follicle, PA–preantral follicle, bar = 100 μm. Tabular data presentation for quantitative analysis of corpora lutea numbers from 2- and 6-month-old WT and Kiss1-PgrKO mice; ^*a*^ represents a difference between 2- and 6-months old WT mice, *p* = 0.024; ^*b*^ represents a difference between 6-month-old WT and Kiss1-PgrKO mice, *p* = 0.026. Data represent means ± SD.

The Kiss1-PgrKO mice exhibited a biphasic reproductive phenotype. The estrous cycling pattern of Kiss1-PgrKO mice were not different from their WT littermates at 2 months of age (Fig 2A), but when examined at the age of 6 months, they either completely lost cyclicity or displayed aberrant cycling patterns (Fig 2A). Similarly, the basal serum LH concentration of Kiss1-PgrKO mice was not different from that of WT controls at the age of 2–3 months (*p* = 0.26). However, when measured at the age of 6 months, Kiss1-PgrKO mice serum LH concentration (0.12 ng/ml) was significantly lower than that of WT control mice (0.46 ng/ml, *p* = 0.024; Fig 2C). Not surprisingly, a similar age-dependent fertility was observed. At 3.5 months of age, the Kiss1-PgrKO mice were already less fertile than the WT controls (22% vs. 66% % in WT; *p* = 0.058) (Fig 2B), but fertility further decreased by 5 months of age (14% vs. 71% in WT; *p* = 0.03) (Fig 2B). At this age, the Kiss1-PgrKO mouse ovary had significantly fewer number of corpora lutea (2.6 ± 1.51) than WT mice (9.4 ± 5.03) (*p* = 0.026) (Fig 2D).

### Kisspeptin PGR is required for the hypothalamic response to estradiol stimulation

**Fig 3:**
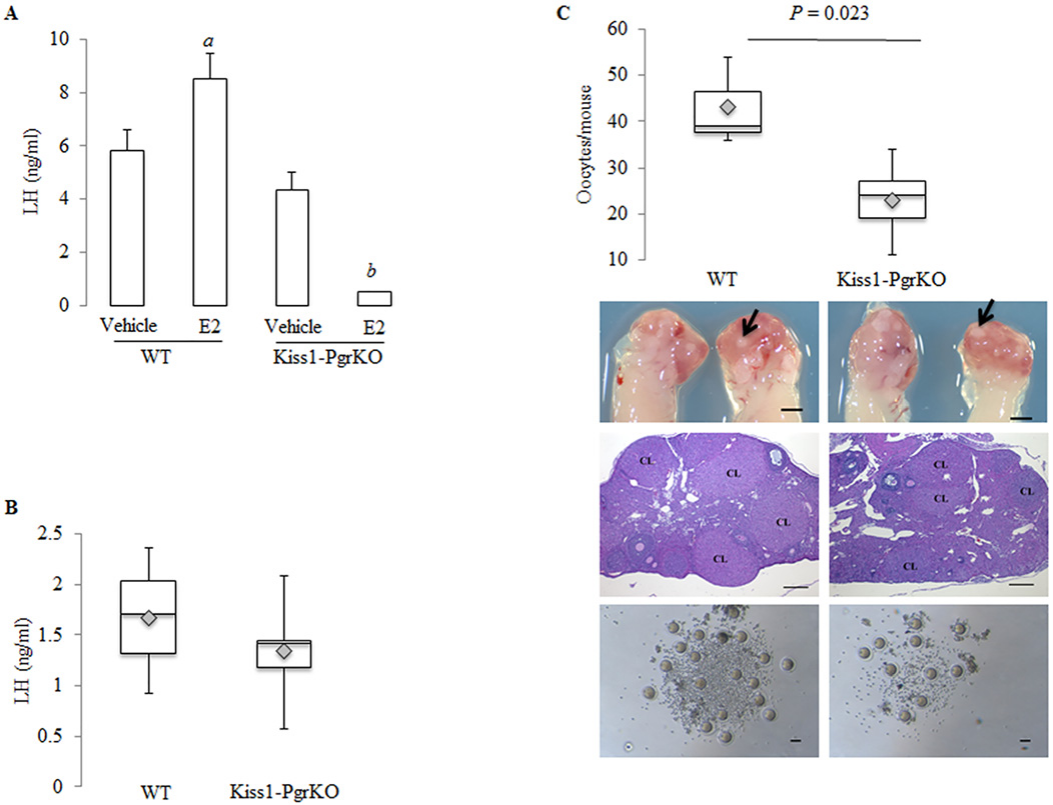
Kiss1-PgrKO mice have impaired kisspeptin-mediated GnRH release. A. Serum LH before and after stimulation of kisspeptin neurons by exogenous administration of estradiol in 6-8-month-old ovariectomized WT and Kiss1-PgrKO mice (n = 3); *a* represent statistical difference between WT, and *b* represents statistical difference between Kiss1-PgrKO mice (*p* = 0.037 and *p* = 0.01, respectively). B. Serum LH concentration 10 min after GnRH stimulation in 5-month-old WT (n = 3) and Kiss1-PgrKO (n = 6) mice (*p* = 0.47). C. Gross morphology, histology, oocyte morphology, and box plot data representation of oocytes from 5-month-old WT (n = 3) and Kiss1-PgrKO (n = 6) mice after ovarian hyperstimulation with gonadotropins; grossly corpora lutea are small, round, tan, nodular structures that can be seen bulging from the ovarian surface (black arrows), and histologically corpora lutea are characterized as multiple large, round, nodular structures that expand the ovarian cortex (*CL*–corpora lutea). Gross image scale bar = 1 mm, histology scale bar = 100 μm, oocyte scale bar = 250 μm.

A robust GnRH discharge from GnRH neurons is required for the LH surge that triggers ovulation [29]. In intact animals, the ovulatory GnRH discharge is initiated in response to elevated circulating estradiol level. A similar type of GnRH secretion can be experimentally induced by injecting a bolus of estradiol to estrogen-primed ovariectomized mice [29]. To test if PGR in kisspeptin neurons is required for the estradiol-triggered induction of GnRH secretion, adult (6–8 months old) WT and Kiss1-PgrKO mice were ovariectomized and primed with estradiol via Silastic capsules. Upon stimulation by estradiol injection, serum LH concentrations rose in WT mice (5.82 ± 0.77 to 8.49 ± 0.7 ng/ml), but dramatically decreased in Kiss1-PgrKO mice (4.33 ± 0.67 to 0.5 ± 0.01 ng/ml; *p* = 0.01) (Fig 3A), whereas serum FSH concentration was similar before and after stimulation in WT and Kiss1-PgrKO mice (data not shown).

The responsiveness of the pituitary to GnRH impacts LH secretion [30, 31]. To ensure that the observed reproductive phenotype of Kiss1-PgrKO mice was not due to a defective pituitary response to GnRH, the pituitary responsiveness of Kiss1-PgrKO mice to GnRH was assessed. Five-month-old WT and Kiss1-PgrKO mice were injected with GnRH and then their serum LH concentrations were measured afterwards. No difference in serum LH concentrations was seen between Kiss1-PgrKO (1.34 ± 0.49 ng/ml) and WT (1.66 ± 0.71 ng/ml) mice (*p* = 0.47) (Fig 3B). Moreover, the number of LH-positive gonadotrophs in the pituitary of Kiss1-PgrKO and WT mice was not different (S1 Fig).

Gonadotropin injections are recognized for many years as a method to induce development of supernumerary follicles and ovulation in mice [32]. To ensure that the observed reproductive phenotype of Kiss1-PgrKO mice was not due to a defective ovarian response to gonadotropins, 5-month-old WT and Kiss1-PgrKO mice were injected with PMSG and hCG following a superovulation protocol [26]. Upon the superovulation induction, both WT and Kiss1-PgrKO mice ovulated a normal number of oocytes. Interestingly, however, a smaller number of oocytes were ovulated from Kiss1-PgrKO than WT mice (23 ± 8.63 in Kiss1-PgrKO vs. 43 ± 9.64 in WT) (*p* = 0.023; Fig 3C). No difference in oocyte quality, measured by the proportions of the degenerate oocytes (S2 Fig), nor in the proportion of the different stages of follicles, as determined by ovarian histology, was seen between the WT and Kiss1-PgrKO mice (S3 Fig).

### Loss of PGR in the kisspeptin neuron does not affect kisspeptin mRNA or protein expression

**Fig 4:**
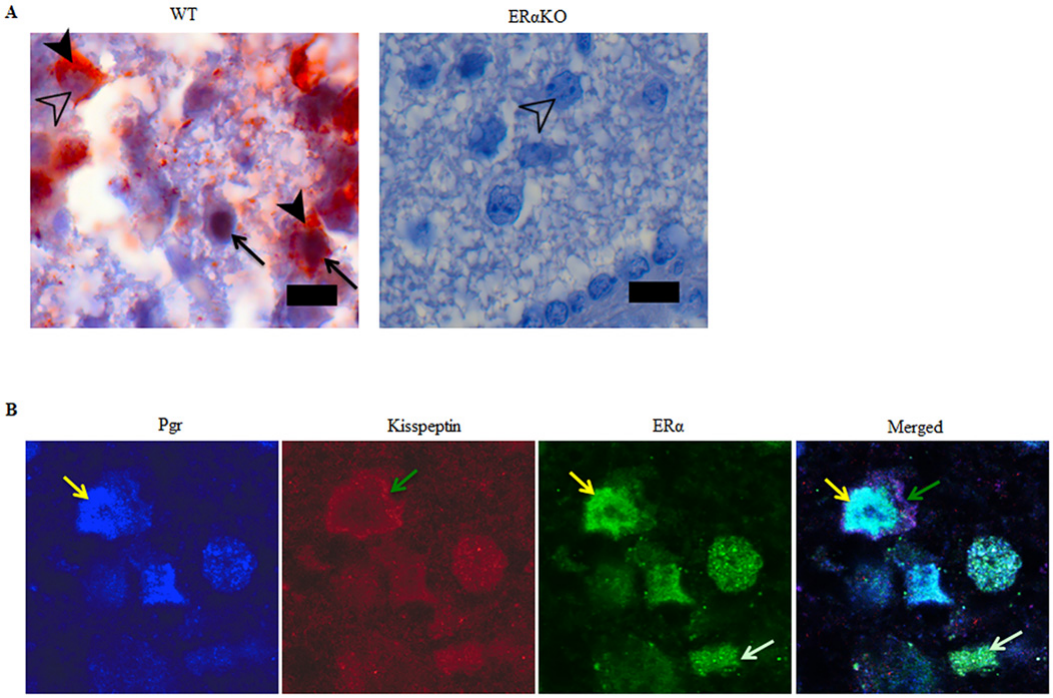
PGR and kisspeptin are co-localized with ERα in kisspeptin neurons but are not expressed in AVPV kisspeptin neurons of ERαKO mice. A. Double immunohistochemistry of PGR and kisspeptin in WT and ERαKO mice. Black arrows indicate dark brown nuclear PGR staining. Black arrowheads indicate brown red cytoplasmic kisspeptin staining. Open arrowhead indicates hematoxylin-stained nucleus of a kisspeptin positive and PGR negative neuron. Bar = 25 μm. B. Co-localization of PGR, ERα, and kisspeptin in kisspeptin neuron in the hypothalamic AVPV nucleus from a 2-month-old female C57BL/6 mouse in the estrus stage. From left to right: PGR (blue), kisspeptin (red), ERα (green), and merged image. Yellow arrows indicate nuclear PGR, ERα, and merged PGR/ERα. Green arrows indicate cytoplasmic kisspeptin. White arrows indicate ERα positive nucleus.

Estradiol regulates GnRH release, in part, by inducing *Kiss1* expression in the kisspeptin neurons via its nuclear receptor transcription factor, ERα [11, 13]. It was therefore determined if PGR plays a role as an ERα downstream mediator in regulating kisspeptin expression. We first determined if PGR expression is under ERα regulation in the kisspeptin neurons by examining PGR expression patterns in the hypothalamus of ERα knockout (ERαKO) mice. PGR expression was not seen in ERαKO AVPV hypothalamic neurons (Fig 4A), suggesting that PGR is an ERα-regulated gene in the kisspeptin neurons as is in other tissues [14–16]. Consistent with this result, PGR expression level in the hypothalamus of WT mice was high in proestrus when circulating estradiol reaches a peak and was low in diestrus when estradiol level is similarly low [33, 34] (S4 Fig).

**Fig 5:**
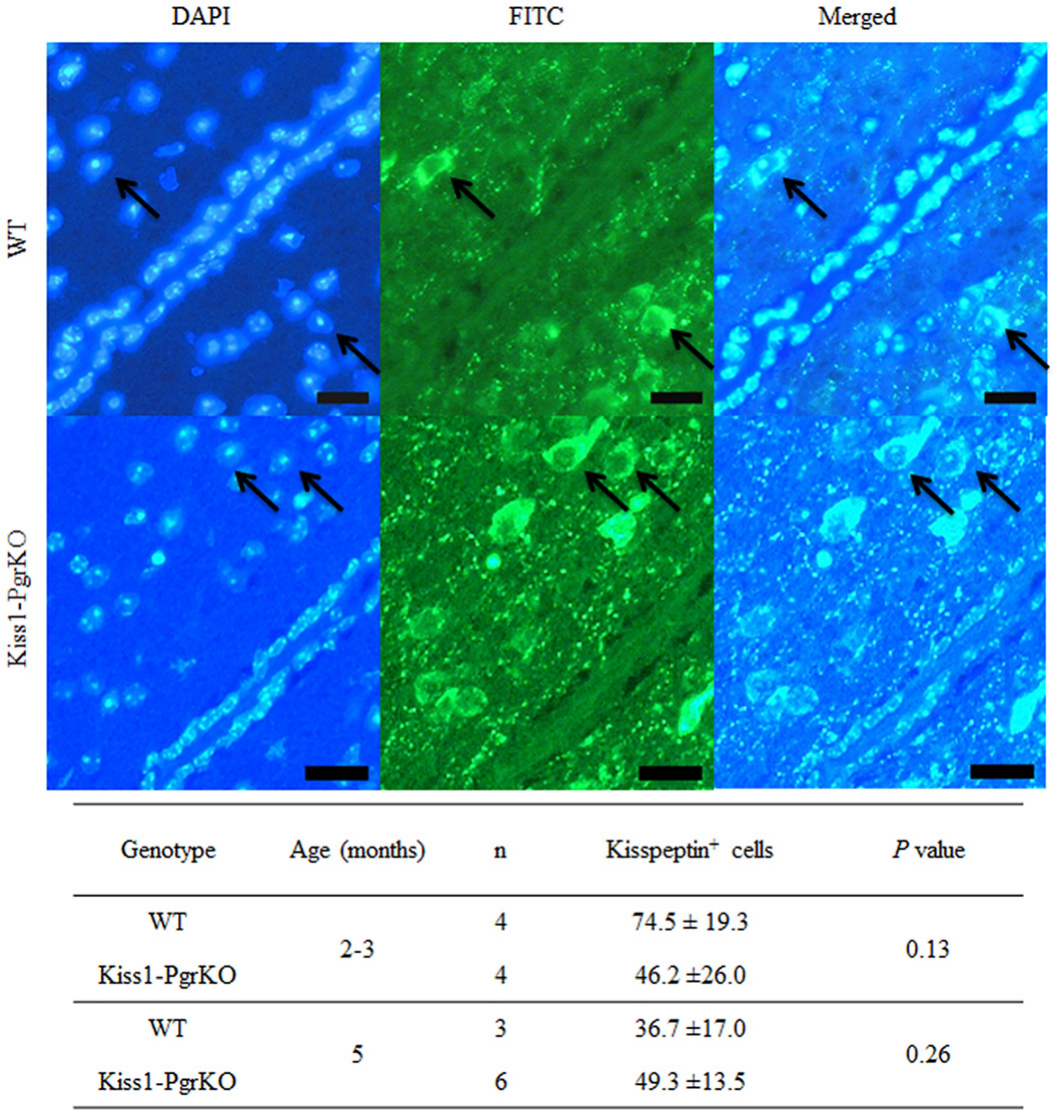
Quantification of kisspeptin positive immunofluorescent cells in the AVPV of WT and Kiss1-PgrKO mice. A. Immunofluorescence of kisspeptin in the AVPV of 5-month-old WT and Kiss1-PgrKO; arrows indicate kisspeptin positive cells (bar = 25 μ). B. Quantification of the raw cell number of kisspeptin positive cells from the AVPV of 2-3- and 5-month-old WT and Kiss1-PgrKO mice; the raw cell number was calculated from 3 sections per animal. Data represent means ± SD.

**Fig 6:**
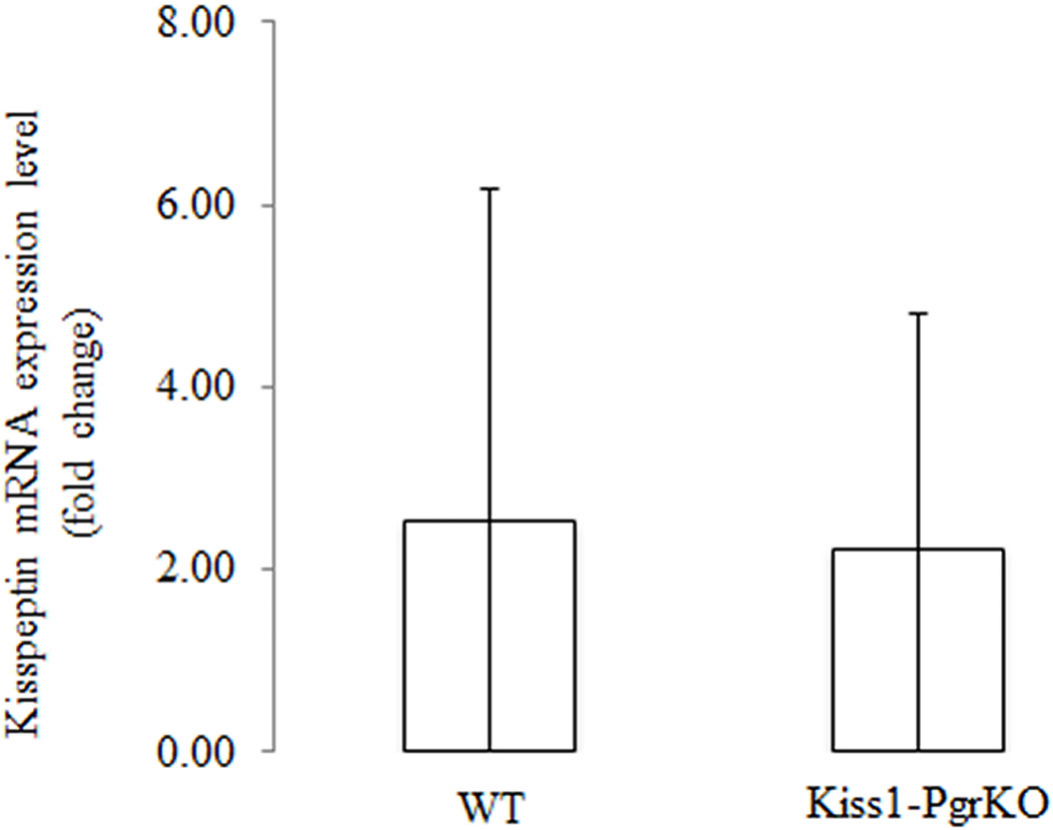
PGR does not regulate *Kiss1* gene expression. *Kiss1* mRNA expression level in 6-month-old WT (n = 9) and Kiss1-PgrKO (n = 4) mice.

Interestingly, when the hypothalami of the ERαKO and Kiss1-PgrKO mice were immunolabeled with anti-kisspeptin antibody, the staining patterns were exceedingly different. Kisspeptin expression was not seen in the hypothalami of ERαKO mice, whereas the Kiss1-PgrKO hypothalami exhibited kisspeptin immunoreactivity that was not different from WT controls (Figs 4 and 5). Kisspeptin positive cell numbers were not different between 2–3 month old WT mice (74.5 ± 19.3 per 0.1 mm^2^) and Kiss1-PgrKO mice (46.2 ± 26.0 cells per 0.1 mm^2^) (*p* = 0.131) or between 5 month-old WT mice (36.7 ±17.0 cells per 0.1 mm^2^) and Kiss1-PgrKO mice (49.3 ± 13.5 cells per 0.1 mm^2^) (*p* = 0.259; Fig 5). Similarly, *Kiss1* mRNA expression level in the AVPV from 6–7 month old WT and Kiss1-PgrKO mice (WT, 2.5 ± 3.6 AU; KO, 2.2 ± 2.6 AU) was not different (Fig 6).

## Discussion

In contrast to the estrogenic regulation of GnRH secretion by ERα, the site and mechanism of GnRH regulation by progesterone are less clear. Expression of PGR in hypothalamic AVPV neurons has been found to be necessary for the estrogenic induction of the preovulatory LH surge [35]. However, the responsible neurons have not been identified, nor has the mechanism of progesteronic regulation of GnRH release been elucidated. In this study, we aimed to determine if kisspeptin neurons are the target of progesterone action in regulating GnRH release. The reproductive consequences of the selective ablation of PGR gene in the kisspeptin neurons indeed reveal that PGR plays a critical role in the kisspeptin neuron in regulating GnRH secretion. In support of this conclusion, we provide compelling evidence that the fertility (Fig 2B), estrous cyclicity (Fig 2A) and ovulation were all severely impaired (Fig 2D) in the mice that are deficient of PGR expression in kisspeptin neurons while the pituitary and the ovary retained their functionality (Fig 3B and 3C). Our findings regarding hypothalamic dysfunction and subsequent reduced fertility are consistent with a recent report from Stephen and colleagues [36]. Nevertheless, our study provides additional insights regarding the absence of LH surge despite normal pituitary response to GnRH, and decreased ovulatory capacity in response to stimulation by exogenous gonadotropins, which opens up a need for an investigation into the consequence of constitutive hypothalamic dysfunction on the ovary.

While it is not yet known how PGR regulates GnRH secretion, our study indicates that PGR likely does so by acting as a downstream mediator of estradiol/ERα action in kisspeptin neurons. In support, in this study it is shown that PGR expression is regulated by ERα in the kisspeptin neurons, as demonstrated by the absence of PGR expression in AVPV neurons of ERαKO mice as well as by a cyclic change in PGR expression level in the hypothalamus (S4 Fig) that is parallel to estradiol level during the estrous cycle of WT mice [33, 34]. These findings were expected because PGR expression is also regulated by estrogen via ERα in other target organs of progesteronic regulation such as the uterus and the central nervous system [14–16]. Of note, no trace of PGR expression was seen in the entire hypothalamus of the ERαKO mouse where PGR is also expressed in cells other than kisspeptin neurons (Fig 1), indicating that ERα may regulate PGR expression in other neurons as well. In addition, unlike in WT controls [29], serum LH level was not elevated in the Kiss1-PgrKO mice upon LH-surge-inducing protocols of E2 treatment but rather it was decreased. It is therefore suggested that the ability of estradiol to induce LH surges is dependent on presence and activation of PGR [28]. It is well established that estradiol via ERα induces *Kiss1* expression [12, 13]. However, it is unlikely that PGR plays a role in regulating *Kiss1* expression as a downstream mediator of estradiol/ERα action because kisspeptin mRNA as well as protein level in the AVPV of Kiss1-PgrKO mice were not different from those of WT controls (Figs 5 and 6). Taken together, these results suggest that PGR may regulate the machinery that governs kisspeptin secretion rather than the kisspeptin synthesis.

If or how PGR regulates kisspeptin secretory machinery or other cellular event(s) remains to be determined. Due to the well-recognized technical difficulty in measuring kisspeptin or GnRH secretion *in vivo*, this challenging, but intriguing, question may require an *in vitro* assay system to be answered. A neuronal cell line, such as the mouse hypothalamic cell line N6 [37], that expresses kisspeptin may serve as an in experimental model for such purpose. Alternatively, kisspeptin secretion patterns in the Kiss1-PgrKO mouse may be compared with those of WT controls using their hypothalamic tissue slices/explants by employing a novel fast scan cyclic voltammetry that was recently developed and successfully applied to quantitatively measure GnRH release by Dr. Moenter’s laboratory [38]. Lack of kisspeptin secretion or significantly impaired secretion in Kiss1-PgrKO hypothalamic slices will indicate a critical role of PGR in regulating kisspeptin secretion.

Interestingly and unexpectedly, the ablation of the *Pgr* gene in kisspeptin neurons did not induce significant fertility defects in the early pubertal life (<3 months old) but did only after they reached the age of 4 months or older (Fig 2B), even though the Cre-mediated deletion of the *Pgr* gene was completed by 2–3 months of ages (Fig 1). We do not know the exact cause of this biphasic fertility phenotype, however, it is likely that it may take a certain time period for the gene deletion effect to become functionally apparent. Of note, a similar age-dependent biphasic fertility phenotype was seen in our previous study where theca cell-specific ERα knockout mice were fertile up to 4 months of age, and then became completely infertile by the age of 6 months [26].

Kiss1-PgrKO mice successfully ovulated following induction of ovulation by administration of exogenous gonadotropins (Fig 3C), demonstrating that loss of PGR in kisspeptin neurons did not affect ovarian responsiveness to gonadotropins. Unexpectedly, however, it was noted that Kiss1-PgrKO mice ovulated significantly fewer oocytes than control mice. If the ovary of the Kiss1-PgrKO mouse had a follicular or oocyte pool that is similar in size and distribution to those of WT controls, no difference in the numbers of ovulated oocytes is expected when stimulated by exogenous gonadotropins for superovulation induction. The reason that less oocytes were released from the mice that were deficient of PGR in kisspeptin neurons is presently unknown, but it is proposed that Kiss1-PgrKO mice had a smaller recruitable pool of follicles that can respond to gonadotropins. During initial recruitment, follicles depart from the resting pool, and initiate growth [39]. This phase of growth takes several weeks and is gonadotropin-independent [39]. The cyclic recruitment phase of follicles is the gonadotropin-dependent stage [40]. In this stage, FSH recruits a cohort of antral follicles for further growth [41]. Therefore, a possible explanation for the reduced numbers of ovulated oocytes observed in Kiss1-PgrKO mice is that these mice exhibit chronic deactivation of the hypothalamic-pituitary-ovarian axis, as indicated by significantly lower serum LH concentration (Fig 2C). This might have reduced the initial recruitment of follicles so that fewer follicles were available for cyclic recruitment when exogenous gonadotropins were administered. In support of this reasoning, hypophysectomized mice supplemented with estradiol and FSH have higher numbers of primary (single layer of granulosa cells) or secondary follicles (2–5 layers of granulosa cells) compared to hypophysectomized non-treated control mice [42]. The suggested mechanistic explanation in that study was that antral follicles aromatize theca-derived androgens to estradiol that is required for the early stages of follicular growth [42]. Therefore, in the current study, prolonged deprivation of the ovaries from adequate level of gonadotropins could have resulted in decreased production of estradiol that in turn affected the initial recruitment of follicles.

In summary, this study found that PGR has a critical functional role in kisspeptin neurons in regulating GnRH secretion, most likely by modulating kisspeptin secretion via a molecular mechanism that is yet to be discovered.

## Supporting Information

S1 Fig Pituitary gonadotrophs in WT and Kiss1-PgrKO. Immunohistochemical labeling of LHβ in pituitary glands from 4–6 months old WT (n = 5) and Kiss1-PgrKO (n = 8) mice, and graphic data presentation of the cumulative raw cell number of LHβ positive cells from three 400X fields (*p* = 0.089). Inset: higher magnification of immunolabeled gonadotrophs. Low magnification image bar = 100 μm. Inset image bar = 50 μm.
(TIF)

S2 Fig Atretic oocytes in WT and Kiss1-PgrKO. Box plot data presentation for the number of atretic oocytes retrieved from 5-month-old WT (n = 3) and Kiss1-PgrKO (n = 6) mice after PMSG and hCG ovarian hyperstimulation.
(TIF)

S3 Fig Ovarian follicular development in WT and Kiss1-PgrKO. Box plot data representation of percent primordial (A), primary (B), preantral (C) and antral (D) follicles from total follicular counts from 2- and 6-month old WT and Kiss1-PgrKO mice (n = 5).
(TIF)

S4 Fig PGR expression in kisspeptin neurons during the estrous cycle. Tabular data presentation for PGR and kisspeptin co-expression in the hypothalamic AVPV nucleus at proestrus, estrus, and diestrus from 2-3-month-old naturally cycling WT mice. *DP*–double positive cells; ^***a***^–difference between proestrus and estrus, *p* = 0.007; ^***b***^–difference between estrus and diestrus, *p* = 0.041; ^***c***^–difference between proestrus and diestrus, *p* = 0.017; *the raw cell number was calculated from 3 sections per animal. Data represent means ± SD.
(TIF)
