# Supplementary figures and images for: Loss of Fertility in the Absence of Progesterone Receptor Expression in Kisspeptin Neurons of Female Mice
Source: PLoS One. 2016 Jul 21;11(7):e0159534. doi: 10.1371/journal.pone.0159534 (PMC4956300; doi:10.1371/journal.pone.0159534)

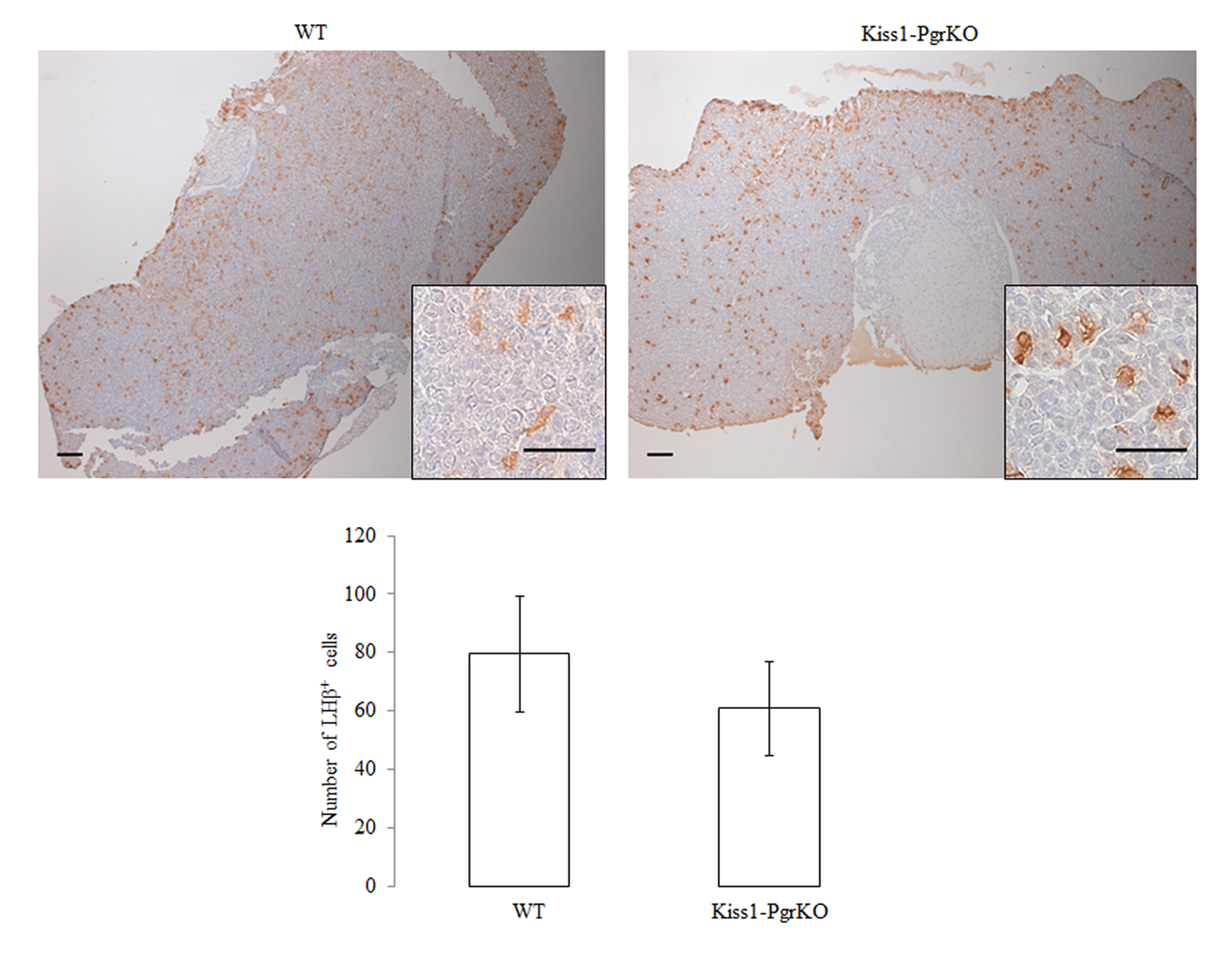

Supplement: S1 Fig — Immunohistochemical labeling of LHβ in pituitary glands from 4–6 months old WT (n = 5) and Kiss1-PgrKO (n = 8) mice, and graphic data presentation of the cumulative raw cell number of LHβ positive cells from three 400X fields (p = 0.089). Inset: higher magnification of immunolabeled gonadotrophs. Low magnification image bar = 100 μm. Inset image bar = 50 μm. (TIF) [file pone.0159534.s001.tif]

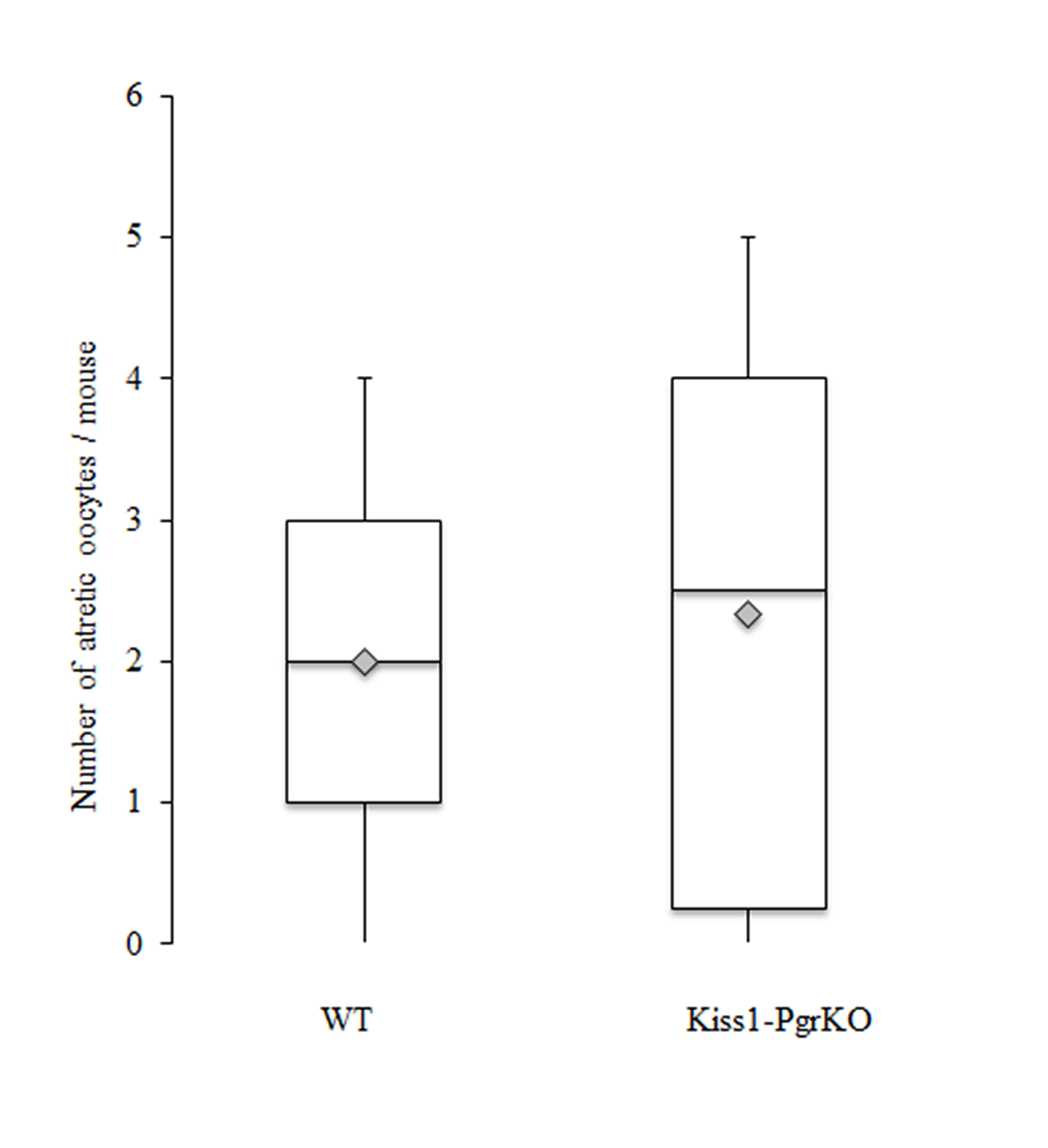

Supplement: S2 Fig — Box plot data presentation for the number of atretic oocytes retrieved from 5-month-old WT (n = 3) and Kiss1-PgrKO (n = 6) mice after PMSG and hCG ovarian hyperstimulation. (TIF) [file pone.0159534.s002.tif]

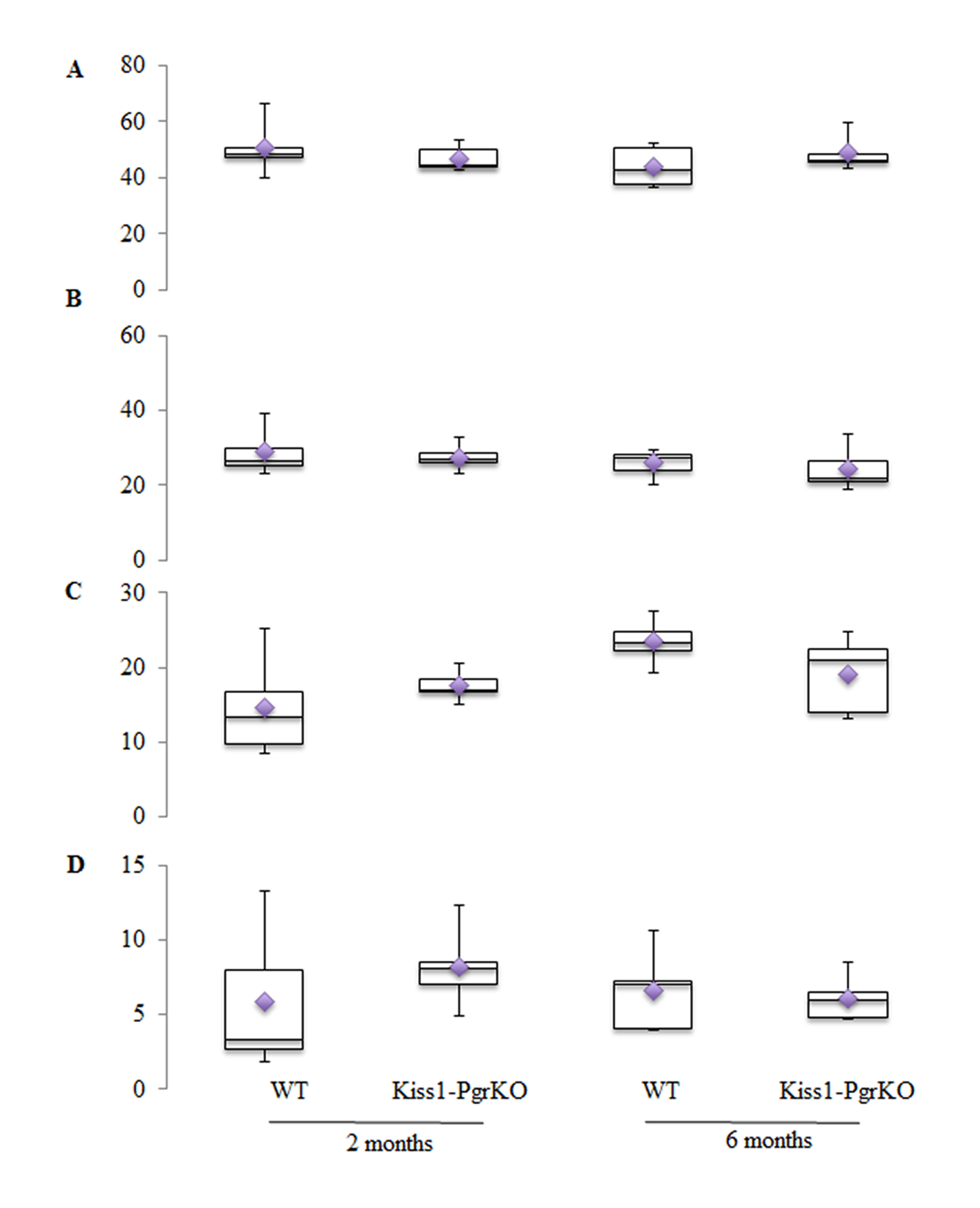

Supplement: S3 Fig — Box plot data representation of percent primordial (A), primary (B), preantral (C) and antral (D) follicles from total follicular counts from 2- and 6-month old WT and Kiss1-PgrKO mice (n = 5). (TIF) [file pone.0159534.s003.tif]

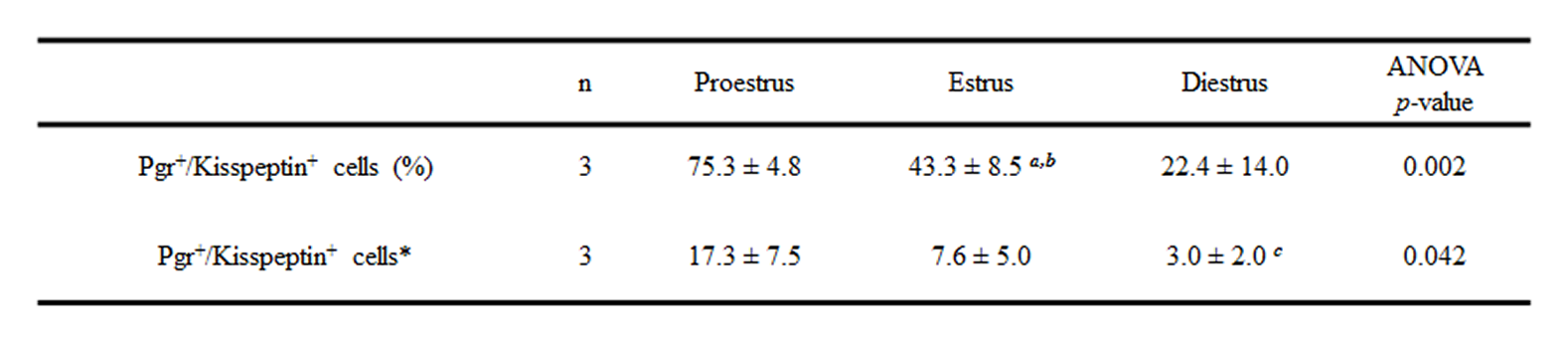

Supplement: S4 Fig — Tabular data presentation for PGR and kisspeptin co-expression in the hypothalamic AVPV nucleus at proestrus, estrus, and diestrus from 2-3-month-old naturally cycling WT mice. DP–double positive cells; a–difference between proestrus and estrus, p = 0.007; b–difference between estrus and diestrus, p = 0.041; c–difference between proestrus and diestrus, p = 0.017; *the raw cell number was calculated from 3 sections per animal. Data represent means ± SD. (TIF) [file pone.0159534.s004.tif]
